# Home medication management problems and associated factors among psychiatric patients using home care pharmacy services at government hospitals in western Malaysia

**DOI:** 10.1186/s12913-022-08069-0

**Published:** 2022-06-01

**Authors:** Christine Li Ling Lau, Cheah Yen Hor, Siew Ting Ong, Muhammad Fadhlullah Roslan, Xin Yi Beh, Dashnilatha Permal, Shamini Rama

**Affiliations:** 1 Pharmacy Department, Bahagia Ulu Kinta Hospital, Ministry of Health Malaysia, Jalan Besar, 31250 Tanjung Rambutan, Perak Malaysia; 2 Pharmacy Department, Seri Manjung Hospital, Ministry of Health Malaysia, Seri Manjung, Perak Malaysia; 3 Pharmacy Department, Teluk Intan Hospital, Ministry of Health Malaysia, Teluk Intan, Perak Malaysia; 4 Pharmacy Department, Raja Permaisuri Bainun Hospital, Ministry of Health Malaysia, Ipoh, Perak Malaysia; 5 Pharmacy Department, Taiping Hospital, Ministry of Health Malaysia, Taiping, Perak Malaysia; 6 Pharmacy Department, Slim River Hospital, Ministry of Health Malaysia, Slim River, Perak Malaysia

**Keywords:** Pharmacy, Home care services, Psychiatry, Pharmacists, Medicine, Hospitals, Malaysia

## Abstract

**Background:**

Proper home medication management plays a role in improving medication adherence, preserving drug efficacy and ensuring safe medication practices, which is crucial to establish positive treatment outcomes. However, no published studies are available on home medication management among psychiatric patients. The study aimed to identify home medication management problems among psychiatric patients in Malaysia and to examine the associations of inappropriate medication storage and lack of a medication administration schedule with sociodemographic factors, disease insight, number of medications and type of home care pharmacy services (HCPS).

**Methods:**

This multicentre cross-sectional study was conducted among psychiatric patients using HCPS in six government hospitals in western Malaysia. Data were extracted from the HCPS form used for each visit as per the protocol published by the Pharmaceutical Services Division, Ministry of Health Malaysia. A minimum sample size of 169 was needed. Proportional random sampling was applied. The associations of inappropriate medication storage and lack of medication administration schedule with study parameters were analysed using multiple logistic regressions.

**Results:**

A total of 205 home visits were conducted with 229 home medication management problems identified; inappropriate medication storage and lack of medication administration schedule topped the list. Inappropriate medication storage was significantly associated with low income [AOR = 4.34 (95% CI 1.17:15.98), *p* = 0.027], alcohol consumption [AOR = 14.26 (95% CI 1.82:111.38), *p* = 0.011], poor insight [AOR = 2.34 (95% CI 1.08:5.06), *p* = 0.030] and part-time HCPS [AOR = 2.60 (95% CI 1.20:5.67), *p* = 0.016]. Lack of administration schedule was significantly associated with low income [AOR = 6.90 (95% CI 1.46:32.48), *p* = 0.014], smoking [AOR = 2.43 (95% CI 1.20:4.92), *p* = 0.013], poor insight [AOR = 5.32 (95% CI 2.45:11.56), *p* < 0.05] and part-time HCPS [AOR = 2.96 (95% CI 1.42:6.15), *p* = 0.004].

**Conclusions:**

Inappropriate medication storage and a lack of a medication administration schedule are common among psychiatric patients. The study also highlighted the potential of HCPS to improve disease insight and home medication management among psychiatric patients if the service is utilized fully.

## Introduction

The overall drug expenditures in 2018 and 2019 by the Malaysian government were USD 1.45 and USD 1.75 billion, respectively [1]. Despite the increasing amount of money spent each year, many patients still failed to achieve their targeted treatment goal. A national survey on the use of medication by Malaysian consumers in 2015 reported that despite extensive use of pharmaceuticals, 18.6% of respondents did not fully understand how to properly use their medication, and 17.0% had no knowledge regarding proper medication storage [2]. Underutilization and nonadherence coupled with poor knowledge of medication management could be contributing factors to treatment failure and wasted resources [3].

The number of patients who have been seeking treatment at government facilities with regard to mental health problems has been increasing steadily over the years. Local statistics from the Malaysian Burden of Disease and Injury Study 2013 showed that in addition to diabetes mellitus and asthma, mental illness was one of the leading causes of nonfatal disease and injury burden [4]. Furthermore, drug-related issues among patients with mental illness can be escalated, further attributable to the chronicity of the illness itself due to the decline in cognitive and executive functioning. The rates of adherence are low for patients with schizophrenia and bipolar affective disorders (50–60 and 35%, respectively) [5]. Additionally, patients with major psychiatric disorders with medication nonadherence can experience exacerbation of their illness and complications, which lead to rehospitalization, poor psychosocial outcomes, relapse of symptoms, reduced effectiveness of subsequent treatment, wasted health care resources, increased substance abuse, poor quality of life and increased suicide [5]. Medications are most often neglected due to stigma, poor family support, inadequate knowledge and the impairment caused by the progression of the illness. Appropriate management of medication is crucial for positive treatment outcomes in this group of patients.

While nonadherence to medication, polypharmacy and inappropriate prescribing has always been a concern in regard to treatment outcomes, it is now recognized that there is a broader range of drug-related problems that need to be addressed. The United States Pharmacopeia classifies medication use processes as prescribing, transcribing/documenting, dispensing, administration and monitoring [6]. While the medication use process in an inpatient setting involves systematic interprofessional collaboration, patients at home must rely on themselves or lay caregivers who lack the necessary skills and knowledge for the drug administration process. This often leads to poor home medication management, such as overstocking of medication, inappropriate medication storage, polypharmacy, use of discontinued medication, expired medication and the use of over-the-counter medication, which are not suitable for their conditions, as well as the lack of a written medication list and instruction to aid timely and correct administration of medicines (i.e., the medication administration schedule) [7].

Studies conducted on other groups of chronically ill patients have reported the rate of inappropriate home medication management to be as high as 57% [5, 8, 9]. Thompson et al. found that the majority of patients kept their medication in the kitchen. In addition, 4 and 8.3% of patients were found to store their medication in the bathroom and at multiple locations, respectively. Many also stored their medication in containers other than the original dispensed packaging, and these containers were not labelled accordingly. The most common reasons cited include portability of medication, the need to split the tablets that were originally blister packs, a dislike of the original packaging and difficulty with opening the original containers. An even more worrisome finding was that approximately 9% of respondents mixed multiple prescription drugs in the same containers [10].

The factors associated with poor medication management may include low health literacy, confusion over the drug name, inability of the patients to read the storage and drug use instructions or unaffordability of patients to their own refrigerators that are required for storage of certain drugs [11, 12]. There are very limited data on the ability of patients with mental illness as well as their caregivers to manage their medications. Many of these problems are complex or even impossible to identify over the counter during drug dispensing, and the way to shed light on these problems is to access patients’ medication at their homes. Thus, as pharmacists conduct home care pharmacy services (HCPSs) for patients with mental illness, it is vital to understand the magnitude of poor home medication management, to identify possible factors attributable to the problem and to help patients and their caregivers better manage their medication at home. Good home medication management practices, such as keeping the medication in an appropriate container, not overstocking medicines, avoiding inappropriate polypharmacy, avoiding expired medicines and having a written medication administration schedule, are important to improve medication compliance, avoid mistakes, prevent overdosing and, most importantly, reduce waste [8, 13].

The HPCS program, previously known as the Home Medication Review, is a collaborative effort with the Community Mental Health Team (CMHT), a service program provided under the Mental Health Act of 2001 and Mental Health Regulations of 2010. The multidisciplinary team comprises various disciplines, including psychiatrists, medical officers, assistant medical officers, registered nurses, pharmacists, occupational therapists and social welfare officers working together to provide comprehensive mental health care services to the community [14]. The pharmacist’s role is to support the team by conducting home medication reviews (rebranded to home care pharmacy services), thus providing education on the importance of treatment of medication, regimens, doses as well as side effects of medication and their management. The pharmacist is also responsible for identifying any medication-related problems and performing the necessary interventions [14]. Patients were selected based on inclusion and exclusion criteria provided in the Psychiatry and Mental Health Services Operational Policy, Ministry of Health Malaysia. The inclusion criteria included patients with severe mental illness, history of nonadherence to treatments, frequent crises, frequent readmissions, prolonged psychotic episodes, and comorbid substance use; the exclusion criteria included primary substance use disorder, personality disorders (such as antisocial personality), and homelessness [14]. Home visits by pharmacists are conducted together with the CMHT multidisciplinary team after obtaining consent from the patient and family, which is done by the case managers prior to patient recruitment into the CMHT program [15, 16].

Earlier studies have concentrated on the adherence rate and other medication-related problems faced by psychiatric patients, but none have focused on home medication management among this group of patients. Education given to patients usually focuses on knowledge regarding their illness, medication and side effects as well as the importance of adherence to their prescribed regimens. Little or no information is disclosed to patients regarding proper home medication management. Nevertheless, this is an important factor that affects a patient’s treatment outcome, as proper home medication management ensures that drug efficacy is maintained, medication safety is ascertained and adherence to medication is improved. Therefore, this study aimed to identify home medication management issues and their associated factors among psychiatric patients using the HCPS program at government facilities in the state of Perak, Malaysia. This study also aimed to identify the associations of inappropriate storage and lack of medication administration schedule with (i) sociodemographic factors, (ii) the number of drugs prescribed, (iii) disease insight, and (iv) the type of HCPS. By identifying these problems and studying the possible associated factors, we hope to clarify the management of medication-related issues in this group of patients and further improve the quality of the HCPS program in Malaysia.

## Methodology

This study was reported in accordance with the STROBE guidelines.

### Study design and sample

This was a multicentre cross-sectional study conducted among patients using the HCPS programme from October 2019 until June 2020 in six government hospitals in the state of Perak, Malaysia. The study was initially planned to last for six months but had to be extended due to the COVID-19 pandemic, which hindered home visiting activities. These six hospitals were chosen because they have at least one pharmacist actively conducting the HCPS program in their respective settings.

All home care pharmacists involved had received certification for their specialization in Pharmacy Psychiatry in HCPS. In accordance with the HCPS Protocol 2nd edition 2019 published by the Pharmaceutical Services Division Malaysia, the HCPS pharmacists were required to report findings such as medication reconciliation, medication storage and medication-related problems using the HCPS-6 form. In practice, the assessment of insight is one component of the mental status examination, an essential tool that aids physicians in making psychiatric diagnoses and is based on clinical judgement. The doctors, registered nurses and assistant medical officers assessed the patient’s awareness and understanding of illness and need for treatment, the degree to which the patient understands how the illness has impacted their life, their relationship with others and their willingness to change by asking questions about the patient during their interview [17, 18]. Information from the HCPS-6 form, Psychiatric Case Report and assessment of insight by the multidisciplinary team was extracted into a data collection form developed by the investigators [14]. The data collection form was pretested to ensure that all relevant data were collected.

Sampling was performed in accordance with the 2018 statistics from the Pharmacy Management Report, whereby a total of 712 psychiatric patients were using the HCPS program in Perak. The intended study duration was six months; thus, the estimated population was taken from half of the total number of patients from 2018, which was 356 patients. Using the sample size calculator for prevalence studies developed by Naing et al. (2006), the precision level was set at 5% and the confidence level was set at 95%, and the minimum sample size required was determined to be 169 [19]. This was based on the assumption that overall home medication management was inappropriate for 70% of patients with chronic diseases [20]. Proportional random sampling was used in the study whereby the number of samples required from each centre was divided proportionally depending on the total number of patients from each setting. A Microsoft Excel (Microsoft Corporation 2010, US) formula was then applied to generate unique random numbers, which were then used to generate a randomized list based on the setting’s patient registry under HCPS.

No missing data were reported in this study, as approaches were taken to avoid any missing data. First, investigators reviewed and fully understood the work process of the CMHT, which the pharmacists work closely with by going to home visits with the team. In accordance with the Community Mental Health Team Service Program Guidelines 2016 (only Malay version available), each patient using the HCPS is assigned to a case manager, which consists of a registered nurse or an assistant medical officer with post basic psychiatric. The case manager is responsible for obtaining the patient’s biodata as accurately and completely as possible to manage the patient, obtain as much family background as possible and make appointments with the patient or family [21]. All information regarding the patient will be documented inside the patient’s Psychiatric Case Report, and this information will be extracted by the pharmacists into the HCPS-6 form and subsequently into the data collection form. The Ministry of Health Malaysia has also implemented ‘Audit of Accuracy and Completeness of Clinical Documentation and the Accuracy of the ICD-10 Code Setting’ (only Malay version available), which is done yearly to ensure complete accurate information has been documented [22]. If the pharmacists found any missing data, they referred back to the case manager to fill in the missing data. In addition, training was conducted for all study personnel.

The study protocol was approved by the National Medical Research Register, Ministry of Health Malaysia, with the registration number 19–2303-49,656. The investigators applied for waivers to obtain informed consent from the patients, as only routine activities were carried out during visits, and no new information was collected from the patient and caregivers. All data collected were kept confidential, and no unique identifiers were collected. The data presented did not identify individuals. Subjects were not given access to their personal information and study data. Data used for publication did not review any identity of the subjects.

### Definition of the study terms

In the study, appropriate home medication management included the appropriate storage of medications, having a drug administration schedule, the absence of medication duplication, the absence of drug hoarding, keeping expired medicines and not sharing medication with others. Appropriate storage was defined as storing medication in a cool, dry place (preferably not in bathrooms or near the kitchen stove) or refrigerator and out of direct sunlight. Medication must be stored to ensure that they cannot be taken by children if the household has children. Medications sensitive to humidity should be stored in their original containers or blisters. For patients using pill boxes, medication removed from blisters should not undergo any changes in physical appearance. Medication duplication is considered present if the patient has two or more medications in the home containing the same active substance or drugs of the same therapeutic class, which was not intended by the prescribing doctor for their chronic illnesses (e.g. two or more painkillers found at home or two different brands of the same medication). The medication administration schedule was defined as having a medication identification chart containing instructions for medication use, such as taking before or after a meal, and the time of administration, which can assist patients in taking medication correctly and in a timely manner [14]. The concept of disease insight was defined by three dimensions: (i) the recognition that one has a mental illness (awareness), (ii) the ability to relabel unusual mental events (delusions and hallucinations) as pathological (attribution) and (iii) the recognition of the need for treatment (action) [23]. In the study, a person was deemed to have poor insight when he indicated unawareness, misattribution and inaction, either alone or together. Monthly household income groups were categorized into three categories, which were the bottom 40% (B40), middle 40% (M40) and top 20% (T20), based on the income thresholds provided by the Department of Statistics Malaysia in 2014 [24]. Full-time HCPS was considered when the pharmacist’s essential duty was to conduct home medication reviews on all working days. Part-time HCPS was considered when the pharmacist’s primary job was to perform their respective outpatient or inpatient department duties on a daily basis and only conduct home medication reviews on selected working days as their secondary job.

### Data analysis

Descriptive statistics were used to describe patients’ sociodemographic characteristics. The normality of continuous data was assessed using the Kolmogorov–Smirnov test. As data were not normally distributed, the median with interquartile range was used. Categorical variables are presented as percentages. Inappropriate medication storage and lack of medication administration schedule were selected for multiple logistic regression analysis. An initial univariate analysis of the studied variables was performed against the patients’ demographic characteristics and the type of HCPS. Only factors that reported a *p* value of 0.25 and below were selected for multivariate analysis. A *p* value of less than 0.05 was considered statistically significant. All statistical analyses were conducted using IBM SPSS Version 20.0 (IBM Corp., Armonk, NY).

## Results

During the study duration, home care pharmacists at participating hospitals conducted a total of 205 home visits. Out of the total number of patients, 54.6% (*n* = 112) were under part-time HCPS, and 45.4% (*n* = 93) were under full-time HCPS. The numbers of male and female patients in the study were similar, with a median age of 45 years (*IQR* = 38–56). The majority of patients were of Malay ethnicity (*n* = 118; 57.6%), followed by Chinese (*n* = 60; 29.3%), Indian (*n* = 19; 9.3%) and others (*n* = 8; 3.9%), which consisted of indigenous people and Sikhs. The median disease duration and number of medications were 14 years (*IQR* = 6–23) and 3 (*IQR* = 2–4), respectively. The level of education of respondents varied widely, from no formal education to tertiary education, with more than half completing secondary schooling (*n* = 120; 58.5%). The majority of patients were unemployed (*n* = 155; 75.6%). Most of the patients were in the B40 group of income earners (*n* = 181; 88.3%) (https://www.dosm.gov.my). None were classified under the T20 group of income earners. The majority of respondents were nonsmokers (*n* = 133; 64.9%), did not consume alcohol (*n* = 195; 95.1%) and had no history of substance abuse (*n* = 180; 87.8%). More than two-thirds (*n* = 157; 76.6%) of respondents had good insight regarding their mental illness (Table 1).

**Table 1:** Demographic characteristics of the study patients (*N* = 205)

Characteristics (***N*** = 205)		Median (***IQR***)	N	%
Age		45 (38–56)	–	–
Disease duration		14 (6–23)	–	–
Number of medications		3 (2–4)	–	–
Gender	Male	–	102	49.8
	Female	–	103	50.2
Ethnicity	Malay	–	118	57.5
	Chinese	–	60	29.3
	Indian	–	19	9.3
	Others	–	8	3.9
Highest Level of Education	No formal education	–	29	14.2
	Primary school	–	42	20.5
	Secondary school	–	120	58.5
	Tertiary education	–	14	6.8
Marital status	Married	–	39	19.0
	Single	–	127	62.0
	Divorced	–	23	11.2
	Widowed	–	16	7.8
Employment	Employed	–	50	24.4
	Unemployed	–	155	75.6
Household Income	B40	–	181	88.3
	M40	–	24	11.7
	T20	–	0	0.0
Smoking	Yes	–	72	35.1
	No	–	133	64.9
Alcohol intake	Yes	–	10	4.9
	No	–	195	95.1
Substance abuse	Yes	–	5	2.4
	No	–	180	87.8
	Has stopped	–	20	9.8
Psychiatric disease	Schizophrenia	–	179	87.2
	Major Depressive Disorder	–	2	1.0
	Bipolar Mood Disorder	–	12	5.9
	Others	–	12	5.9
Presence of comorbidities	Yes	–	81	39.5
	No	–	124	60.5
Insight	Good	–	157	76.6
	Poor	–	48	23.4

Home care pharmacists identified a total of 229 home medication management problems. Of these, 31.9% (*n* = 73) were classified as inappropriate storage. Four major categories were found: inappropriate location, medication not in the original container, multiple storage areas for medication and multiple medications in the same container. Other home medication management problems identified included drug hoarding (*n* = 41, 17.9%), not having a medication administration schedule (*n* = 71, 31.0%), sharing of medication (*n* = 4, 1.7%) and the presence of expired medication at home (*n* = 8, 3.5%). Patients were found to keep medication that had already been discontinued by their doctors and were not taking their medication according to a standard repeated frequency and timing daily. There was no medication duplication found, but the home care pharmacists identified the possibility of medication duplication (*n* = 32, 14.0%) in the patients’ homes (Table 2).

**Table 2:** Types of home medication management problems identified during home visits, *N* = 229*

Types of home medication management problems	n (%)
Inappropriate storage of medication	73 (31.9)
Drug hoarding	41 (17.9)
No medication administration schedule	71 (31.0)
Possible medication duplication	32 (14.0)
Sharing of medication	4 (1.7)
Presence of expired medication at home	8 (3.5)

*Observation was only performed once for each patient at one visit. A patient may have more than one home medication problem, but the same problem is not repeated (e.g.. in a condition where a patient has several medications, it will be considered as one problem regardless of the number of medications that were inappropriately stored)

The results of the multivariate logistic regressions are shown in Table 3 and Table 4. Compared to Malays, Indians were associated with an 84% reduction in the odds of having inappropriate storage of medication at home [AOR = 0.16 (95% CI 0.03:0.84), *p* = 0.031]. Patients under the B40 household income category have higher odds (four times more likely) of having inappropriate storage of medication at home versus patients under the M40 household income category [AOR = 4.34 (95% CI 1.17:15.98), *p* = 0.027]. In addition, patients who consumed alcohol were fourteen times more likely to have inappropriate storage of medication at home than patients who did not consume alcohol [AOR = 14.26 (95% CI 1.82:111.38), *p* = 0.011]. Patients who had poor insight into their disease and treatment were two times more likely to have inappropriate storage of medication at home [AOR = 2.34 (95% CI 1.08:5.06), *p* = 0.030]. Furthermore, patients enrolled under part-time HCPS were twice as likely to report inappropriate storage of medication at home than those who were under full-time HCPS [AOR = 2.60 (95% CI 1.20:5.67), *p* = 0.016] (Table 3).

**Table 3:** Logistic regression of factors associated with inappropriate storage of medications at home (*N* = 205)

Variables	Univariate Analysis				Multivariate Analysis			
	Crude OR	95% CI of OR	Wald’s χ^**2**^(df)	***p*** value	Adjusted OR	95% CI of OR	Wald’s χ^**2**^(df)	***p*** value
**Gender**								
Male	0.72	(0.29, 1.77)	0.50 (1)	0.478				
Female	1.00							
**Age (year)**	1.03	(0.99, 1.06)	3.57 (1)	***0.059***	1.03	(0.99, 1.06)	3.32 (1)	0.068
**Ethnicity**			5.82 (3)	***0.120***			6.27 (3)	0.099
Chinese	1.33	(0.56, 3.14)	0.44 (1)	0.506	1.38	(0.62, 3.06)	0.64 (1)	0.423
Indian	0.16	(0.03, 0.86)	4.54 (1)	***0.033***	0.16	(0.03, 0.84)	4.65 (1)	0.031*
Others	1.34	(0.21, 8.56)	0.09 (1)	0.753	1.34	(0.23, 7.67)	0.11 (1)	0.738
Malay	1.00				1.00			
**Highest level of education**			1.47 (3)	0.689				
No formal education	0.96	(0.20, 4.61)	0.03 (1)	0.959				
Primary school	0.51	(0.11, 2.27)	0.77 (1)	0.380				
Secondary school	0.78	(0.20, 2.94)	0.13 (1)	0.718				
Tertiary education	1.00							
**Marital status**			2.66 (3)	0.446			2.51 (3)	0.473
Single	1.47	(0.57, 3.79)	0.65 (1)	0.420	1.44	(0.58, 3.59)	0.63 (1)	0.427
Divorced	2.27	(0.63, 8.07)	1.60 (1)	***0.205***	2.37	(0.68, 8.28)	1.85 (1)	0.173
Widowed	0.67	(0.15, 3.03)	0.25 (1)	0.611	0.78	(0.19, 3.17)	0.11 (1)	0.735
Married	1.00				1.00			
**Employment**								
Unemployed	0.89	(0.39, 2.05)	0.06 (1)	0.793				
Employed	1.00							
**Household Income**								
B40	4.20	(1.12, 15.74)	4.53 (1)	***0.033***	4.34	(1.17, 15.98)	4.87 (1)	0.027*
M40	1.00				1.00			
**Smoking**								
Yes	1.28	(0.47, 3.48)	0.24 (1)	0.624				
No	1.00							
**Alcohol intake**								
Yes	16.55	(1.90, 143.66)	6.48 (1)	***0.011***	14.26	(1.82, 111.38)	6.42 (1)	0.011*
No	1.00				1.00			
**Substance abuse**			0.04 (2)	0.977				
Yes	1.28	(0.11, 14.72)	0.04 (1)	0.838				
Ex-abuser	1.09	(0.32, 3.67)	0.02 (1)	0.887				
No	1.00							
**Total number of medications**	1.23	(0.98, 1.55)	3.30 (1)	*0.069*	1.23	(0.98, 1.54)	3.40 (1)	0.065
**Insight**								
Poor	2.37	(1.07, 5.22)	4.61 (1)	***0.032***	2.34	(1.08, 5.06)	4.72 (1)	0.030*
Good	1.00				1.00			
**HCPS**								
Part-time	2.46	(1.07, 5.65)	4.51 (1)	***0.034***	2.60	(1.20, 5.67)	5.85 (1)	0.016*
Full-time	1.00				1.00			

* *p* value below 0.05 is considered statistically significant; *CI* confidence interval, *OR* odds ratio

**Table 4:** Logistic regression of factors associated with lack of medication administration schedule at home (*N* = 205)

Variables	Univariate Analysis				Multivariate Analysis			
	Crude OR	95% CI of OR	Wald’s χ^**2**^(df)	***p*** value	Adjusted OR	95% CI of OR	Wald’s χ^**2**^(df)	***p*** value
**Gender**								
Male	1.70	(0.66, 4.36)	1.23 (1)	0.267				
Female	1.00							
**Age (year)**	1.01	(0.97, 1.04)	0.56 (1)	0.451				
**Ethnicity**			0.35 (3)	0.949				
Chinese	1.28	(0.50, 3.27)	0.27 (1)	0.597				
Indian	1.30	(0.37, 4.60)	0.17 (1)	0.676				
Others	1.15	(0.17, 7.69)	0.02 (1)	0.879				
Malay	1.00							
**Highest level of education**			4.34 (3)	***0.227***			4.71 (3)	0.194
No formal education	0.34	(0.06, 1.76)	1.62 (1)	***0.202***	0.33	(0.07, 1.55)	1.94 (1)	0.163
Primary school	0.85	(0.19, 3.79)	0.04 (1)	0.836	0.85	(0.21, 3.42)	0.04 (1)	0.826
Secondary school	0.41	(0.10, 1.59)	1.64 (1)	***0.199***	0.42	(0.11, 1.53)	1.71 (1)	0.190
Tertiary education	1.00				1.00			
**Marital status**			2.52 (3)	0.472				
Single	0.85	(0.31, 2.32)	0.09 (1)	0.765				
Divorced	2.01	(0.53, 7.64)	1.07 (1)	0.301				
Widowed	0.67	(0.13, 3.30)	0.23 (1)	0.629				
Married	1.00							
**Employment**								
Unemployed	1.07	(0.45, 2.51)	0.02 (1)	0.871				
Employed	1.00							
**Household Income**								
B40	6.45	(1.29, 32.18)	5.16 (1)	***0.023***	6.90	(1.46, 32.48)	5.98 (1)	0.014*
M40	1.00				1.00			
**Smoking**								
Yes	1.97	(0.73, 5.32)	1.79 (1)	***0.180***	2.43	(1.20, 4.92)	6.12 (1)	0.013*
No	1.00				1.00			
**Alcohol intake**								
Yes	0.77	(0.14, 4.11)	0.09 (1)	0.763				
No	1.00							
**Substance abuse**			0.68(2)	0.711				
Yes	0.41	(0.03, 4.94)	0.48 (1)	0.485				
Ex-abuser	0.65	(0.19, 2.21)	0.46 (1)	0.496				
No	1.00							
**Total number of medications**	0.94	(0.73, 1.21)	0.17 (1)	0.676				
**Insight**								
Poor	5.61	(2.50, 12.59)	17.53 (1)	***p < 0.05***	5.32	(2.45, 11.56)	17.85 (1)	p < 0.05*
Good	1.00				1.00			
**HCPS**								
Part-time	2.88	(1.21, 6.83)	5.79 (1)	***0.016***	2.96	(1.42, 6.15)	8.51 (1)	0.004*
Full-time	1.00				1.00			

* *p* value below 0.05 is considered statistically significant; *CI* confidence interval, *OR* odds ratio

Patients under the B40 income category have higher odds (six times more likely) of not having a medication administration schedule [AOR = 6.90 (95% CI 1.46:32.48), *p* = 0.014] than those under the M40 income category. The odds of not having a medication administration schedule at home were two times higher in smokers versus nonsmokers [AOR = 2.43 (95% CI 1.20:4.92), *p* = 0.013]. Patients with poor insight had higher odds (5 times more likely) of not having a medication administration schedule than patients with good insight [AOR = 5.32 (95% CI 2.45:11.56), *p* < 0.05]. Furthermore, patients who were under part-time HCPS were two times more likely to not have a medication administration schedule at home than those who were under full-time HCPS [AOR = 2.96 (95% CI 1.42:6.15), *p* = 0.004] (Table 4).

## Discussion

To our knowledge, this study is the first to examine home medication management problems in psychiatric patients. Kapplan et al. stated that ethnic minority patients were much more likely to have higher levels of noncompliance, independent of other demographic factors [25]. Furthermore, South Asians in Western countries were also found to be more likely to record nonadherent behaviour to oral medication than white Caucasians [26]. To elaborate, studies have found that language barriers in health care led to miscommunication between health care workers and patients, leading to a compromise in the quality of health care delivery and a threat to patient safety [27, 28]. Tideman et al. also found language barriers to be a long-term problem in Malaysia’s health care system due to the population’s different ethnicities [29]. Therefore, misinterpretation of information on medication storage conditions during counselling sessions is plausible. Interestingly, while other studies showed that ethnic minorities were practising poorer quality medication management, this study showed that Indians, as a minority race, were more likely to have better medication storage practices than Malays. Further studies are needed to identify other possible causes that could have led to this finding.

Second, our study showed that patients from lower household income were more likely to have poorer medication storage practices and a lack of an administration schedule at home. This finding is similar to the finding by Martins et al. that lower household income was a marginally significant predictor of an increased risk of inadequate medication storage, although no explanation was given [30]. In Malaysia, there are approximately 2.7 million households in the B40 income category, of whom 44% are in rural areas and 56% are in urban areas [31]. Poor medication management among this group could be attributed to their living environment, where only bare necessities are available in their homes. Some homes do not have furnishings such as tables and refrigerators to store their medications, and many patients do not own a mobile phone, which could serve as a reminder for their medication schedule. Sadly, some do not even have a working clock at home to tell them the time. Although Boron et al. claimed that compensatory strategies such as associating medication schedules with mealtimes are important to improve adherence, the reality is that some underprivileged households in Malaysia struggle to have three fixed main meals a day [32].

Third, our study found that smokers were more likely to not have a medication administration schedule. Although there is currently no literature available to support any association between smoking and the presence of a medication schedule, many studies have demonstrated smoking to be strongly associated with poorer adherence to medication [33, 34]. As medication schedule serves as an aid for better adherence, a lack of adherence would most likely give rise to unsatisfactory adherence to medication. This could be because smokers, who are already engaged in a chronic unhealthy lifestyle habit, may find it difficult to commit themselves to a fixated medication administration routine that requires a certain degree of discipline. Moreover, there appears to be a general consensus that smokers portray significantly lower quality of life (QoL) in the physical, psychological, social and environmental dimensions of health. Problems of self-care and usual activities were found to be among the highest reported problems among smokers [35]. Self-care is defined as the ability of individuals, families and communities to promote health, prevent disease, maintain health, and cope with illness and disability with or without the support of a health care provider, while the usual activities dimension evaluates the severity of problems in their usual activities, such as work, study, housework and family or leisure activities [36, 37]. Therefore, smokers who are generally incapable of promoting and maintaining their health by themselves, along with difficulty in conducting their daily activities, are expected to fail to adhere to timely medication consumption.

Moreover, this study also revealed that alcohol consumption among patients was strongly associated with inappropriate storage of medications at home, consistent with the result of a secondary study of cohort data by Bryson et al. [38]. This is most likely due to the effect of excessive alcohol consumption, which can impede brain functions and result in cognitive as well as behavioural impairments [39]. The effect of alcoholism on cognitive function coupled with the nature of psychiatric disease itself could have caused direct detrimental consequences on a patient’s drug management, namely, drug storage condition and medication adherence.

In addition, this study also found that poor insight was associated with inappropriate medication storage and lack of medication schedule at home. While there are no studies that have linked poor insight with inappropriate storage of medication as well as lack of medication schedule, previous studies have demonstrated a significant relationship between poor insight and poor adherence as well as poor adherence and inappropriate storage of medication at home. Novick et al. and Misdrahi et al. claimed that patients with a lack of insight into their diseases had a higher risk of nonadherence to their medication [40, 41]. Jimmy et al. also stated that having a medication administration schedule serves as an aid to improve medication adherence [42]. Thus, it is plausible to infer that those without a medication administration schedule are deemed to have poor adherence to their medication. Smaje et al. affirmed that poor medication storage was negatively associated with adherence to medication [43]. A possible explanation for this could be that patients with better insight into their diseases will undeniably have a more positive attitude towards their medication, thus resulting in better medication management practices, such as proper storage of medication, as well as having a medication schedule at home. Therefore, patients with better insight tend to have better medication management practices and thus better medication adherence [44].

Last but not least, part-time HCPS was found to be one of the factors associated with both inappropriate medication storage and lack of medication administration schedule among patients compared to full-time HCPS. These two medication management problems are heavily affected by patient cooperation on a daily basis. Leach MJ affirmed that good rapport with patients essentially leads to a stronger therapeutic alliance with patients and can significantly improve the effectiveness of health care services [45]. Therefore, a good rapport between the HCPS pharmacists and the patient and family is important for gaining their trust and cooperation to practice better medication storage and administration schedules. However, this requires continuous follow-up visits and higher contact time between the pharmacists and the patient as well as their family. Unfortunately, the nature of part-time HCPS makes this rather difficult, which supports the findings of this study. Studies have shown that HCPS can enhance patient understanding, prevent medication accidents and lead to patient benefits if the service is well performed and utilized appropriately [46, 47].

### Recommendation

Overall, our findings indicate that full-time HCPS shows more benefits in regard to patients’ home medication management, which could further contribute to their medication adherence as well. However, there are conflicting findings on the benefits of home-based pharmacy services. A meta-analysis by Abott et al. assessing the impact of pharmacist home visits for individuals at risk of medication-related problems concluded that there were no benefits on the effect of hospital admission or mortality rates, with limited evidence in improving patients’ quality of life [48]. However, this meta-analysis varied considerably in sample populations, focus and goals, and type of intervention when compared to our study, thus possibly contributing to the conflicting findings. Furthermore, Abott et al.’s study involved populations with existing comorbid chronic diseases, elderly patients with mean age ranging from 65 to 86, and patients recently discharged from emergency admissions, while our study population includes patients with mental health issues at younger ages ranging from 38 to 56 [48]. Another study by Tan et al. with a similar study population to ours found that home medication reviews conducted by pharmacists not only positively impact patients’ medication adherence but also improve patients’ knowledge of antipsychotic medications and quality of life among patients with schizophrenia [49]. Thus, raising the question as to how the Ministry of Health Malaysia can further improve this service for the benefit of patients. As one of the key strategies to boost population health is through community-based services, it might be beneficial to give HCPS due attention, as there is room for improvement for the well-being of patients. The roles of pharmacists have shifted towards services-based and patient-centred, but emphasis is usually places on services within health facilities. By expanding these services to patients’ homes, HCPS can help fill the gap, which may hinder the effectiveness and care provided at health facilities. A complete and updated protocol on HCPS is in place, but it is not fully utilized, as the service is usually considered secondary. One strategy is to allow appointed home care pharmacists to concentrate on their service rather than placing more emphasis on counter services. This can be done through better human resource management within the Ministry of Health, as the main reason given deterring the pharmacist from conducting home visits is a lack of manpower. More studies on the benefits of full-time HCPS in countering long-standing nonadherence and poor medication management issues would be helpful to systematically quantify its outcomes both clinically and economically.

### Study limitations

This study has some limitations. First, the Movement Control Order (MCO) was issued during part of the study period in response to the COVID-19 pandemic transmission, leading to disruption in home visits, especially to red zone areas. This might have disrupted the medication refill by the patients, especially during the initial phase of MCO, whereby patients were requested to stay indoors. Some patients were also afraid to come to the hospital for medication refills, causing them to run out of medication during HCPS home visits. Some patients might have adjusted their medication administration schedule, such as from once daily dosing to every alternate day dosing to prolong their medication supply. Although this may not have any effect on medication storage at home, it may have greatly impaired patients’ medication administration schedules during the COVID-19 pandemic.

Second, it has been suggested that to have a successful logistic regression model, the data should contain at least ten events for each variable entered, although the validity of this general principle has been questioned [50]. We decided to include the variables based on a literature review whereby studies had found factors such as gender, age, households with children (may be linked to marital status), educational background, occupation, smoking status and number of medications to be significantly associated with home medication management practices [30, 51, 52]. In addition, we included household income because based on our observation, education background and occupation did not guarantee higher income. Furthermore, we were interested in determining whether ethnicity played a role in home medication management practices, as ethnic minorities are usually exposed to barriers to accessing medication information. However, based on our data and in reference to the rule of thumb, we should have studied fewer than seven predictor variables for inappropriate medication storage. As we included more than the acceptable variables for adjustment in the multivariate logistic regression of factors associated with inappropriate medication storage at home as well as the presence of an administration schedule, the robustness of the statistical methods could have been affected.

In addition, the effect of sociodemographic factors (ethnicity, educational level and income) on patients’ home medication management is rather difficult to explain due to limited studies available and confounding variables, as they may not be truly independent factors influencing medication management.

## Conclusion

This study highlights issues on home medication management among psychiatric patients and possible associated factors. The findings indicate that certain sociodemographic factors, disease insight and the type of HCPS are significantly associated with medication management at home. Nevertheless, the findings shed light on the benefits of full-time HCPS and good disease insight towards patients’ home medication management. Thus, with improved implementation of HCPS as well as thorough patient counselling by pharmacists, home medication management among psychiatric patients may perhaps be improved.

## Data Availability

The datasets used and/or analysed during the current study are available from the corresponding author upon reasonable request and with permission of the Director General of Health Malaysia.

## Abbreviations

HCPS: Home Care Pharmacy Services
CMHT: Community Mental Health Team
B40: Income thresholds below 40%
M40: Income thresholds middle 40%
T20: Income thresholds top 20%
MCO: Movement Control Order
